# Inactivation of TOPK Caused by Hyperglycemia Blocks Diabetic Heart Sensitivity to Sevoflurane Postconditioning by Impairing the PTEN/PI3K/Akt Signaling

**DOI:** 10.1155/2021/6657529

**Published:** 2021-04-23

**Authors:** Sumin Gao, Rong Wang, Siwei Dong, Jing Wu, Bartłomiej Perek, Zhengyuan Xia, Shanglong Yao, Tingting Wang

**Affiliations:** ^1^Department of Anesthesiology, Union Hospital, Tongji Medical College, Huazhong University of Science and Technology, 1277 Jiefang Avenue, Wuhan 430022, China; ^2^Department of Emergency Medicine, The Affiliated Huaian No.1 People's Hospital of Nanjing Medical University, Huai'an, Jiangsu 223001, China; ^3^Department of Cardiac Surgery and Transplantology, Poznan University of Medical Sciences, Poznań, Poland; ^4^State Key Laboratory of Pharmaceutical Biotechnology, The University of Hong Kong, Hong Kong, China; ^5^Department of Anesthesiology, Affiliated Hospital of Guangdong Medical University, Zhanjiang, Guangdong, China

## Abstract

The cardioprotective effect of sevoflurane postconditioning (SPostC) is lost in diabetes that is associated with cardiac phosphatase and tensin homologue on chromosome 10 (PTEN) activation and phosphoinositide 3-kinase (PI3K)/Akt inactivation. T-LAK cell-originated protein kinase (TOPK), a mitogen-activated protein kinase- (MAPKK-) like serine/threonine kinase, has been shown to inactivate PTEN (phosphorylated status), which in turn activates the PI3K/Akt signaling (phosphorylated status). However, the functions of TOPK and molecular mechanism underlying SPostC cardioprotection in nondiabetes but not in diabetes remain unknown. We presumed that SPostC exerts cardioprotective effects by activating PTEN/PI3K/Akt through TOPK in nondiabetes and that impairment of TOPK/PTEN/Akt blocks diabetic heart sensitivity to SPostC. We found that in the nondiabetic C57BL/6 mice, SPostC significantly attenuated postischemic infarct size, oxidative stress, and myocardial apoptosis that was accompanied with enhanced p-TOPK, p-PTEN, and p-Akt. These beneficial effects of SPostC were abolished by either TOPK kinase inhibitor HI-TOPK-032 or PI3K/Akt inhibitor LY294002. Similarly, SPostC remarkably attenuated hypoxia/reoxygenation-induced cardiomyocyte damage and oxidative stress accompanied with increased p-TOPK, p-PTEN, and p-Akt in H9c2 cells exposed to normal glucose, which were canceled by either TOPK inhibition or Akt inhibition. However, either in streptozotocin-induced diabetic mice or in H9c2 cells exposed to high glucose, the cardioprotective effect of SPostC was canceled, accompanied by increased oxidative stress, decreased TOPK phosphorylation, and impaired PTEN/PI3K/Akt signaling. In addition, TOPK overexpression restored posthypoxic p-PTEN and p-Akt and decreased cell death and oxidative stress in H9c2 cells exposed to high glucose, which was blocked by PI3K/Akt inhibition. In summary, SPostC prevented myocardial ischemia/reperfusion injury possibly through TOPK-mediated PTEN/PI3K/Akt activation and impaired activation of this signaling pathway may be responsible for the loss of SPostC cardioprotection by SPostC in diabetes.

## 1. Introduction

Ischemic heart disease is one of the leading causes of death worldwide, particularly in patients with diabetes [1]. Restoration of blood flow to an ischemic heart is necessary to salvage the ischemic myocardium, but reperfusion of an ischemic region can result in myocardial ischemia/reperfusion (IR) injury due to oxidative stress [2]. Studies have shown that sevoflurane postconditioning (SPostC) could protect the myocardium against reperfusion-induced injury and reduce myocardial infarct size [3, 4]. However, patients with diabetes have a remarkably higher mortality rate owing to increased IR injury and the beneficial effects of SPostC almost completely disappeared [5–7]. Therefore, elucidating the underlying mechanisms to prevent the myocardial IR injury in diabetes is a significant challenge faced by modern anesthetic practices.

Phosphatase and tensin homolog deleted on chromosome ten (PTEN) is a dual lipid and protein phosphatase that antagonizes the phosphatidylinositol 3-kinase/Akt (PI3K/Akt) signaling pathway and regulates cellular survival and growth. Studies have shown that cardiac-specific PTEN inactivation protects myocardial IR injury by activating the PI3K/Akt signaling in mice [8]. However, cardiac PTEN is increased in streptozotocin- (STZ-) induced diabetic rats, which is responsible for the loss of diabetic heart sensitivity to ischemic postconditioning through PI3K/Akt inactivation [9]. In addition, accumulating evidence has demonstrated that SPostC protects the rat hearts against IR injury via the activation of the PI3K/Akt signaling, which is one of the important signaling pathways involved in IR injury [10]. These investigations collectively suggest that the PTEN/PI3K/Akt signaling plays an important role in SPostC-induced cardioprotection and impairment of this signaling may be responsible for the loss of SPostC cardioprotection in diabetes. Thus, interventions that can enhance PTEN/PI3K/Akt activation may serve as a promising therapy against hyperglycemia-induced myocardial IR injury.

T-LAK cell-originated protein kinase (TOPK) is a mitogen-activated protein kinase- (MAPKK-) like serine/threonine kinase, which plays a critical role in tumorigenesis and cell cycle regulation [11–13]. TOPK promotes cell proliferation and migration by modulating the PTEN/PI3K/Akt pathway and is associated with poor prognosis in lung cancer [14]. Interestingly, studies have shown that activation of TOPK-mediated antioxidation protects against focal cerebral IR injury [15]. Our previous study has demonstrated that remote ischemic postconditioning protects against renal IR injury that was associated with activation of TOPK and concomitant reduction in oxidative stress and inflammation [16]. However, whether TOPK plays significant roles in myocardial IR remains unclear. Of note, studies have shown that ischemic preconditioning alleviated myocardial IR injury and induced TOPK activation in rats, while TOPK inhibition aggravated the H_2_O_2_-induced oxidative stress in H9c2 cardiomyocytes [17]. These results suggest that TOPK may mediate a novel survival signal in myocardial IR through inhibiting oxidative stress. However, whether TOPK plays roles in SPostC cardioprotection through PTEN/PI3K/Akt and whether the impairment of this signaling is attributable to the loss of SPostC protection on the diabetic myocardium remain unclear.

Therefore, in the present study, TOPK or PI3K/Akt inhibitor and TOPK adenovirus were used both in *in vivo* models of myocardial IR in diabetic mice and *in vitro* models of hypoxia/reoxygenation (H/R) in the embryonic rat cardiomyocytes H9c2 cells, to test the hypotheses that SPostC protected against myocardial IR injury by activation of PTEN/PI3K/Akt through TOPK and that impairment of the TOPK/PTEN/Akt signaling blocked diabetic heart sensitivity to SPostC.

## 2. Materials and Methods

### 2.1. Experimental Animals and the Induction of Diabetes

Male C57BL/6 mice (7-8 weeks old) purchased from Wuhan University Animal Experiment Center (Wuhan, China) were used in this study. All animal studies were approved by the Institutional Animal Care and Use Committee of Tongji Medical College of Huazhong University of Science and Technology, China.

Mice were rendered diabetic by continuous intraperitoneal injection of streptozotocin (STZ) (40 mg/kg; Sigma-Aldrich, Merck Millipore, Darmstadt, Germany) diluted in citrate buffer (pH 4.2-4.5) for 5 days, whereas age- and sex-matched mice were injected with an equivalent volume of vehicle only (citrate buffer, pH 4.2-4.5). Three days after the last injection of STZ, the blood glucose concentrations of all mice were measured twice a week and mice with glucose levels higher than 300 mg/dl were considered as diabetic. All mice were housed in a temperature-controlled room and maintained on standard chow with free access to water. At termination (8 weeks after STZ treatment), mice were weighed and then subjected to myocardial IR as previously described [3].

### 2.2. Animal Experimental Protocol

**Figure 1:**
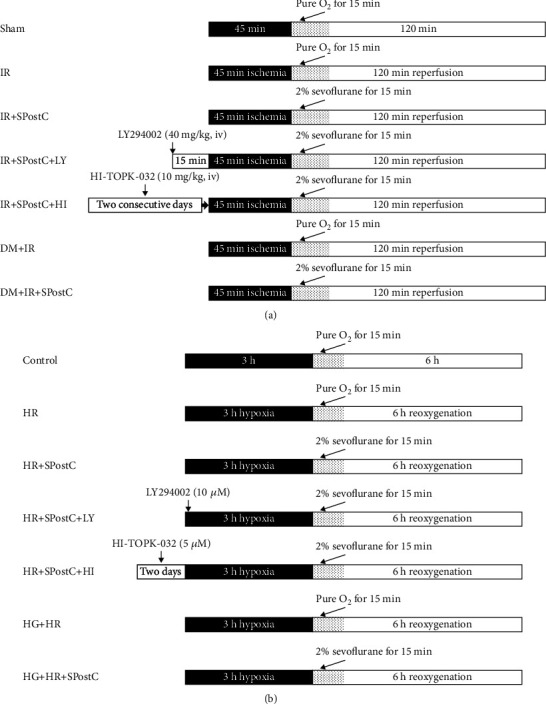
Model flow diagram of sevoflurane postconditioning (SPostC) protective effects against myocardial ischemia/reperfusion (IR) injury. (a) Experimental animal models. (b) Schematic presentation of the cell models.

The mice were randomly divided into seven groups as follows: (1) Sham: nondiabetic C57BL/6 mice received the surgery and were threaded the ligature underneath the left anterior descending coronary artery (LAD); however, there is no actual ligation in LAD; (2) IR: nondiabetic C57BL/6 mice underwent 45 min ischemia, followed by 120 min reperfusion; (3) IR+SPostC: nondiabetic C57BL/6 mice received IR and SPostC treatments. SPostC was achieved via continuous inhalation of 2% sevoflurane during the first 15 minutes of the reperfusion period; (4) IR+SPostC+LY: nondiabetic mice were pretreated with LY294002 (Sigma-Aldrich, Germany), a PI3K inhibitor, and then subjected to IR and SPostC. LY294002 was injected intraperitoneally at a single dose of 40 mg/kg 15 minutes before inducing coronary ischemia [16]; (5) IR+SPostC+HI: nondiabetic mice were pretreated with HI-TOPK-032 (Tocris Bioscience, UK), a TOPK-specific inhibitor which is reported to inhibit TOPK kinase activity [12, 16], and then subjected to IR and SPostC. HI-TOPK-032 was injected intraperitoneally at a single dose of 10 mg/kg for two consecutive days before inducing ischemia [16]; (6) DM+IR: diabetic mice underwent IR as previously described; (7) DM+IR+SPostC: diabetic mice underwent IR and SpostC as previously described. All groups except the IR+SPostC+HI and IR+SPostC+LY group received the same volume of vehicle intraperitoneally. The treatment protocol is outlined in Figure 1(a).

### 2.3. Cell Protocol

Embryonic rat cardiomyocytes H9c2 cells were obtained from the China Center for Type Culture Collection (Wuhan, China). Cells were cultured in Dulbecco's modified Eagle's medium with 5.5 mM (normal glucose) or 25 mM glucose (high glucose) containing 10% (*v*/*v*) fetal bovine serum (FBS, Gibco) and grown at 37°C in a humidified atmosphere of 5% CO_2_ and 95% air. Cells reached 80% to 90% confluence were subjected to experimental procedures.

The H9c2 cells were subjected to hypoxia/reoxygenation (3 h of hypoxia followed by 6 h of reoxygenation) to achieve hypoxia/reoxygenation (HR) model as previously described [3]. The cells were divided into seven groups: (1) control: the H9c2 cells were incubated with 5.5 mM (normal glucose) glucose without performing HR; (2) HR: the H9c2 cells were incubated with normal glucose and subjected to HR; (3) HR+SPostC: H9c2 cells in the SpostC group were placed in a closed container at a temperature of 37°C; sevoflurane evaporation tank was then opened to allow 2% sevoflurane to flow through the closed container with O2 for 15 minutes at the onset of reoxygenation. Sevoflurane and oxygen concentrations were monitored using a multifunctional detector (PM8050; Drägerwerk, Germany) for the same duration of time; (4) HR+SPostC+LY: the H9c2 cells exposed to a normal glucose concentration in the presence of 10 *μ*M LY294002 were subjected to HR and SpostC [18]; (5) HR+SPostC+HI: the H9c2 cells exposed to a normal glucose concentration in the presence of HI-TOPK-032 were subjected to HR and SPostC. HI-TOPK-032 was given at 5 *μ*M for two days before inducing HR [19]; (6) high glucose (HG)+HR: the H9c2 cells were incubated with a high-glucose concentration and subjected to HR; (7) HG+HR+SPostC: the H9c2 cells incubated with a high-glucose concentration were subjected to HR and SPostC. The model is illustrated in Figure 1(b).

The subgroups of H9c2 cells under high glucose were infected with adenoviral vectors encoding for full-length rat TOPK (Ad-TOPK) or adenoviral vectors (Ad-vector) provided by Vigene Bioscience (Jinan, China) for three days (MOI, 50). The generation of full-length rat TOPK was in term of NM_001079937.1 gene from NCBI. Recombinant adenovirus was constructed with a virus titer of 1 × 10^11^ vp/ml. After transfection with Ad-TOPK or Ad-vector, cells were subjected to HR with or without SPostC as described above. An additional group of H9c2 cells transfected with Ad-TOPK was treated with LY294002 before hypoxia stimulation. Following termination of the experiment, the cells and medium were collected and stored at -80°C until analysis.

### 2.4. Determination of Risk and Infarct Sizes

Myocardial infarct size was assessed by the Evans blue/TTC staining after 2 h reperfusion. The unstained region by Evans blue dye was considered as the area at risk (AAR), and the white color area was considered as the infarct size (IS). The area at risk was calculated as a percentage of the total ventricular area, while the infarct size was measured by macroscopic method and the infarcted area reported as the percentage of the area at risk. The extent of the area of necrosis was determined by computerized planimetry (ImageJ 1.4).

### 2.5. Measurement of Creatinine Kinase-MB (CK-MB)

After reperfusion for 2 h, blood samples were collected and the release of CK-MB was measured by enzyme-linked immunoassay using a commercial kit (Uscn Life Science Inc., China) as previously described [2].

### 2.6. Detection of Myocardial Apoptosis and Immunofluorescence

Myocardial apoptosis was determined by TUNEL staining using an in situ cell detection kit (TUNEL assay, Roche Diagnostics GmbH, Mannheim, Germany). Tissues were extracted from infarcted regions of the myocardium. After fixation and permeabilization, TUNEL assays were performed. Tri-formol-fixed myocardial tissue samples embedded in paraffin were detected to identify the apoptotic cells via a fluorescence microscope. The TUNEL-positive cells were counted under a high-power microscopic field at a magnification of 400x, and at least three randomly selected fields per heart were analyzed.

The myocardial sections were incubated overnight with rabbit anti-TOPK (Phospho Thr9) antibody (Abcam, UK) at 4°C. After being washed, the sections were further incubated with CY3 goat anti-rabbit IgG at room temperature for 2 h followed by DAPI staining at room temperature for 10 min. All the stained sections were photographed by using an inverted microscope (Olympus Life Science, Tokyo, Japan) with a color CCD camera.

### 2.7. Measurement of Oxidative Stress

After 2 h reperfusion, blood samples were collected for the measurement of malondialdehyde (MDA) concentration. MDA was determined by using a thiobarbituric acid (TBA) method using a trace assay kit (Nanjing Jiancheng Bioengineering Institute, Nanjing, China) as reported [20]. Superoxide dismutase (SOD) level was measured by hydroxylamine method using an assay kit (Nanjing Jiancheng Bioengineering Institute, Nanjing, China).

Myocardial superoxide anion production was measured by fluorescent-labeled dihydroethidium (DHE) staining (Keygen Biotech Co., Ltd., Nanjing, China) according to the assay kit protocol.

### 2.8. Measurement of Cell Viability and Lactate Dehydrogenase (LDH)

After 6 h reoxygenation, cell viability was determined by the MTT assay (Sigma-Aldrich, Germany) as described, and the supernatant of cell culture was collected for the measurement of LDH through a commercially available detection kit (Nanjing Jiancheng Bioengineering Institute, Nanjing, China) via the colorimetric method according to the manufacturer's instructions.

### 2.9. Western Blot Analysis

Protein extracts were prepared according to the manufacturer's protocol as described in the protein extract kit (Keygen Biotech Co., China). Peri-infarct region of the left ventricular myocardium was harvested after 2 h of reperfusion in order to extract total protein samples for immunoblotting analysis. Total protein was determined using the BCA Protein assay kit (Sigma-Aldrich, Germany), and size was separated by sodium dodecyl sulfate-polyacrylamide gel electrophoresis (SDS-PAGE). Proteins were transferred to PVDF membrane. Primary antibodies were then incubated with the membrane strips at 4°C overnight at the following dilutions: Akt, phospho-Akt (Ser473), PTEN, and phospho-PTEN (Ser380/Thr382/383) (Cell Signaling Technology, USA) 1 : 1000 and TOPK and phospho-TOPK (Thr9) (Abcam, UK) 1 : 1000. Then, protein bands were incubated with a secondary antibody and detected by the ECL chemiluminescence method. Densitometric analyses of western blot images were performed using ImageJ software (ImageJ 1.4).

### 2.10. Statistical Analysis

All the values were expressed as the mean ± SD unless otherwise stated. GraphPad Prism software package (San Diego, CA) was used for data statistical analysis. One-way analysis of variance (ANOVA) was performed to detect significant differences between the experimental groups, followed by a *t*-test corrected for multiple comparisons (Student-Newman-Keuls). The *P* values < 0.05 were considered statistically significant.

## 3. Results

### 3.1. Physiological Parameters

As presented in Table 1, the water intake, food consumption, and blood glucose levels in the mice with STZ-induced diabetes were higher than those in the normal control mice (*P* < 0.001), while body weight was significantly lower in diabetic mice than that in nondiabetic control (*P* < 0.001).

As shown in Table 2, the heart rate and mean arterial pressure were recorded throughout the experimental period. No significant difference was observed in average heart rate among the seven groups at baseline and throughout the experiments. There was no significant difference in the baseline mean arterial pressure among all groups. The mean arterial pressure during ischemia and reperfusion was lower than the baseline in all groups (*P* < 0.01) except the sham group. During myocardial IR, the mean arterial pressure of all diabetic groups was remarkably lower than that of the nondiabetic IR group (*P* < 0.05, Table 2). After 2 h reperfusion, the mean arterial pressure in the IR+SPostC group was increased compared with that in the IR group (*P* < 0.05).

### 3.2. Myocardial Ischemia/Reperfusion Injury *In Vivo*

**Figure 2:**
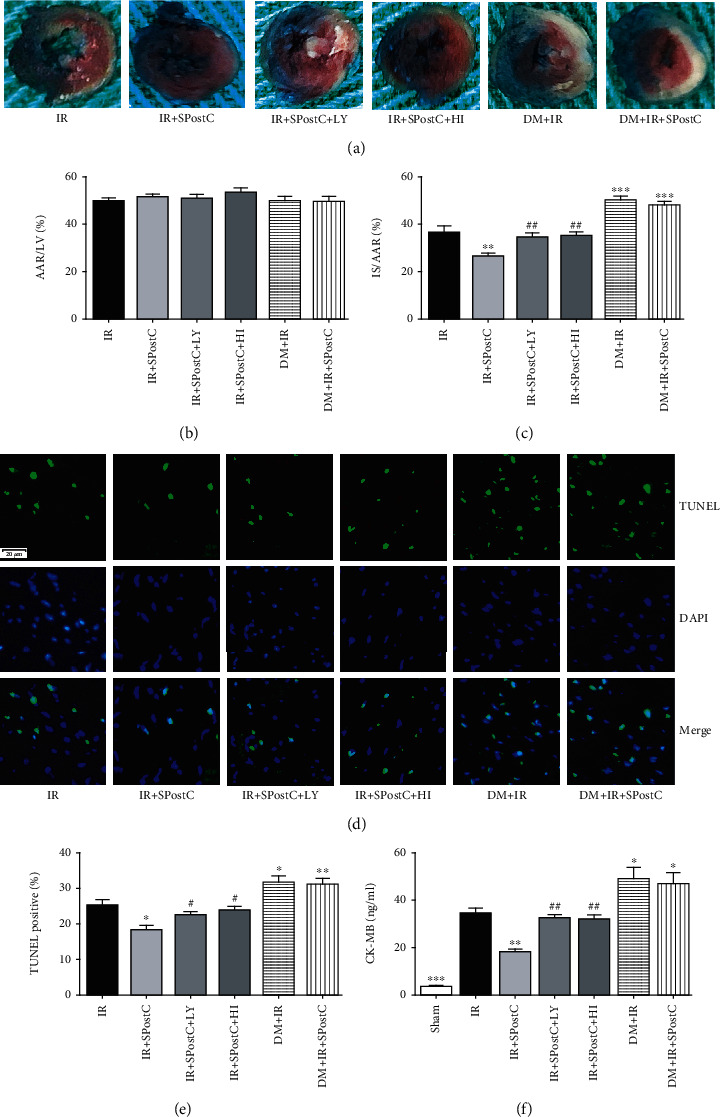
Myocardial IR injury and myocardial apoptosis after 45 min coronary occlusion followed by 120 min reperfusion with or without SPostC in nondiabetic and diabetic mice. IR and SPostC indicated nondiabetic mice received IR or IR+SPostC, respectively; IR+SPostC+LY and IR+SPostC+HI indicated nondiabetic mice pretreated with LY294002 (LY, a PI3K inhibitor) or HI-TOPK-032 (HI, a TOPK kinase inhibitor), respectively, and then subjected to IR and SPostC. DM+IR and DM+IR+SPostC indicated diabetic mice received IR or IR+SPostC, respectively. (a) Representative images of Evans blue and TTC staining in heart cross sections from each experimental group. Infarct area (INF: white); area at risk (AAR: red and white); perfused area (blue). (b) Comparison of area at risk per left ventricle (area at risk/left ventricle). (c) Comparison of area of infarct size normalized to the area at risk (infarct size/area at risk). (d) Myocardial apoptosis was assessed by the TUNEL assay in the heart sections (DAPI: nuclei, blue; TUNEL: apoptosis nuclei, green; magnification, ×400). (e) Quantification of TUNEL-positive cardiomyocytes (% of total). (f) Plasma CK-MB secretion detected using a commercial ELISA kit. All values are presented as the mean ± SD (*n* = 7 per group). ^∗^*P* < 0.05, ^∗∗^*P* < 0.01, and ^∗∗∗^*P* < 0.001 compared with the IR group; ^#^*P* < 0.05 and ^##^*P* < 0.01 compared with the SPostC group.

The area at risk and the infarct size in the sham group were zero in nondiabetic mice (Figure S1(a)), which was consistent with our previous study in rat [21]. As shown in Figures 2(a) and 2(b), the area at risk did not differ significantly among the other IR groups, which meant that our myocardial IR model was reliable and reproducible. As shown in Figure 2(c), SPostC greatly attenuated the infarct size in C57BL/6 nondiabetic mice (*P* < 0.01). However, LY294002 and HI-TOPK-032, respectively, abolished the protective effect of SPostC in increasing infarct size in nondiabetic mice (*P* < 0.01). Diabetes significantly increased the myocardial infarct size in the DM+IR and DM+IR+SPostC group compared with the IR group (*P* < 0.001). However, the anti-infarct effect of SPostC was abolished completely in the diabetic mice (DM+IR+SPostC group vs. DM+IR group, *P* > 0.05).

CK-MB is one of the major biomarkers of myocardial cellular injury and can reflect the damage degree of heart. As shown in Figure 2(f), the release of plasma CK-MB was significantly increased after myocardial IR in nondiabetic mice (*P* < 0.001). SPostC significantly decreased the release of plasma CK-MB when compared with the IR group (*P* < 0.01). The effect of SPostC in decreasing CK-MB secretion was abolished by LY294002 and HI-TOPK-032 (*P* > 0.01). When compared with the nondiabetic groups, diabetes displayed elevated postischemic CK-MB release (DM+IR and DM+IR+SPostC groups vs. IR group, *P* < 0.05). Nevertheless, SPostC exerted no significant effect on the serum level of CK-MB in diabetic mice (DM+IR group vs. DM+IR+SPostC group, *P* > 0.05).

As shown in Figures 2(d) and 2(e), SPostC significantly reduced cardiomyocyte apoptosis, as evidenced by the decreased number of TUNEL-positive myocyte nuclei when compared with the IR group in nondiabetic mice (*P* < 0.05). However, the antiapoptotic effect of SPostC was canceled by LY294002 and HI-TOPK-032 (*P* < 0.05). Diabetic mice displayed increased cardiomyocyte apoptosis, as evidenced by the increased number of TUNEL-positive myocyte nuclei when compared with the nondiabetic groups (DM+IR and DM+IR+SPostC groups vs. IR group, *P* < 0.05). However, SPostC did not exert an antiapoptotic effect in the hearts of diabetic mice (DM+IR group vs. DM+IR+SPostC group, *P* > 0.05).

### 3.3. Levels of TOPK, PTEN, and Akt in the Hearts of Nondiabetic Mice

**Figure 3:**
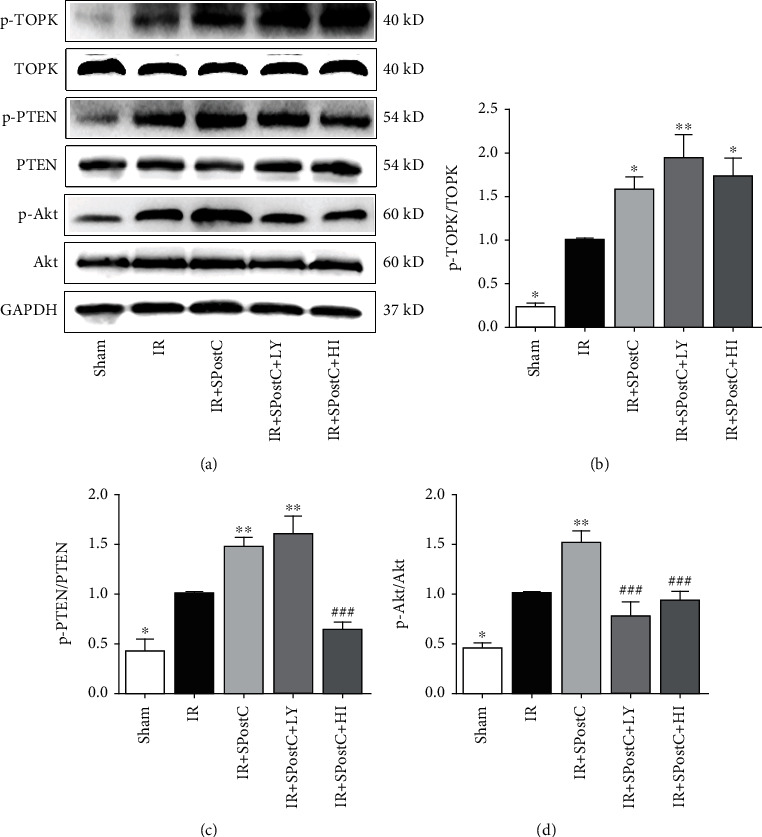
Myocardial TOPK (b), PTEN (c), and Akt (d) protein expression and their phosphorylation status after myocardial IR with or without SPostC in nondiabetic mice. IR+SPostC+LY and IR+SPostC+HI indicated mice pretreated with LY or HI, respectively, and then subjected to IR and SPostC. (a) Representation of western blots. Mean band density was normalized relative to GAPDH. The IR group was used as control and normalized to unity, and the protein expression of other groups was displayed as changes over this baseline. All values are presented as the mean ± SD (*n* = 7 per group). ^∗^*P* < 0.05 and ^∗∗^*P* < 0.01 compared with the IR group; ^###^*P* < 0.001 compared with the SPostC group.

As shown in Figure 3, IR significantly induced the phosphorylation of cardiac TOPK, PTEN (inactivation), and Akt, which was further significantly increased by SPostC treatment (Figure 3, *P* < 0.05). LY294002, a PI3K inhibitor, decreased the Akt phosphorylation induced by SPostC (*P* < 0.05), but had no effect on TOPK or PTEN phosphorylation. Studies have shown that HI-TOPK-032 was docked to the active site of TOPK and directly suppressed TOPK kinase activity, which can inhibit the activation of its downstream target molecules but has no effect on the expression of total and phosphorylated TOPK [12]. As shown in Figure 3, HI-TOPK-032 remarkably blocked the increase of PTEN and Akt phosphorylation induced by SPostC (*P* < 0.05). However, the protein level of total and phosphorylated TOPK did not significantly change in the IR+SPostC+HI group when compared with the IR+SPostC group.

### 3.4. Posthypoxic Cellular Injury and Levels of TOPK, PTEN, and Akt in H9c2 Cardiomyocytes Exposed to Normal Glucose

**Figure 4:**
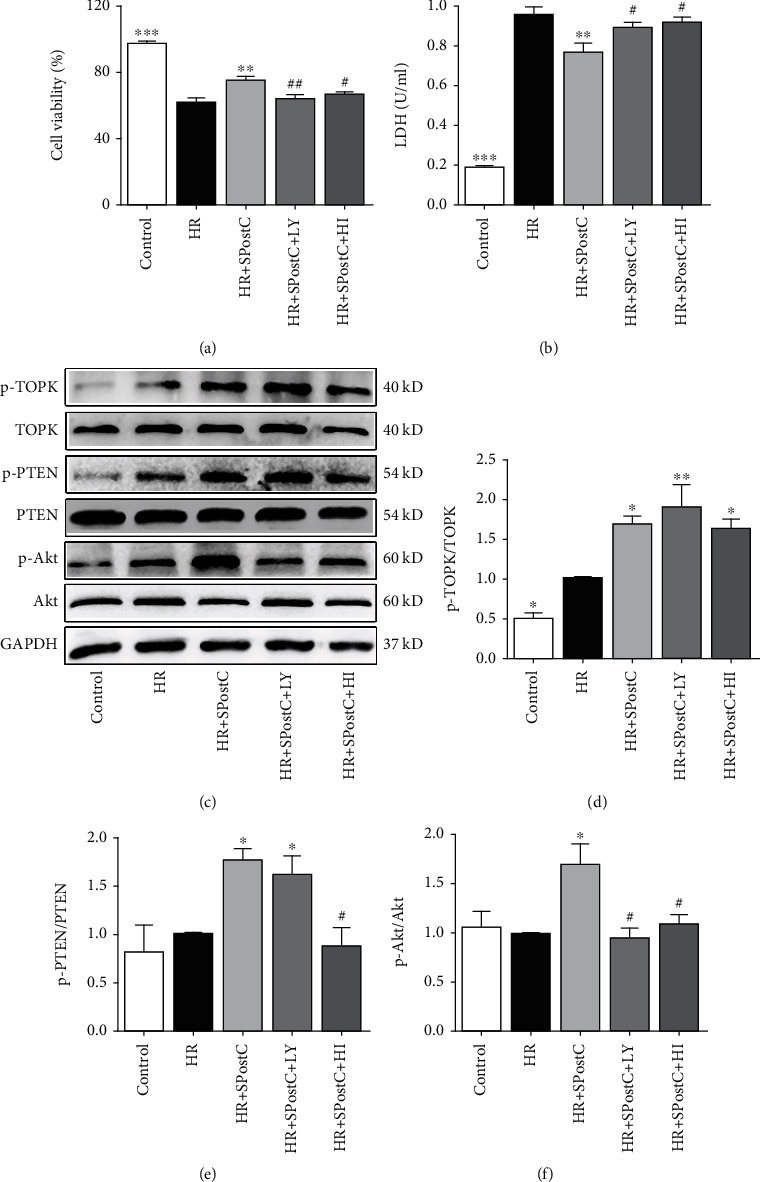
Cardiomyocyte injury and myocardial TOPK, PTEN, and Akt protein expression and their phosphorylation status assessed after hypoxia-reoxygenation (HR) with or without SPostC in H9c2 cells under normal glucose. LY or HI were applied, respectively, to block Akt and TOPK activation in H9c2 cells. (a) Cell viability assessed by MTT assay. (b) Lactate dehydrogenase (LDH) release. (c) Representative western blots. (d–f) Expression of TOPK (d), PTEN (e), and Akt (f) and their phosphorylation status. Mean band density was normalized relative to GAPDH. The IR group was used as control and normalized to unity, and the protein expression of other groups was displayed as changes over this baseline. All values are presented as the mean ± SD of three independent experiments each performed in triplicate. ^∗^*P* < 0.05, ^∗∗^*P* < 0.01, and ^∗∗∗^*P* < 0.001 compared with the HR group; ^#^*P* < 0.05 and ^##^*P* < 0.01 compared with the SPostC group.

Posthypoxic cell death reflected as a decrease in cardiomyocyte viability and an increase in LDH activity in the medium after HR injury (Figures 4(a) and 4(b), *P* < 0.05). SPostC markedly increased cell viability and decreased LDH leakage in normal glucose condition (*P* < 0.05), which was canceled by LY294002 and HI-TOPK-032 in H9c2 cells.

In normal glucose condition, the phosphorylated levels of TOPK, PTEN, and Akt were elevated in the HR+SPostC group compared with the HR group (Figures 4(c)–4(f), *P* < 0.05). HI-TOPK-032 likewise inhibited the phosphorylation of PTEN and Akt induced by SPostC in H9c2 cells (*P* < 0.05). However, LY294002 blocked the phosphorylation of Akt induced by SPostC (*P* < 0.05), but had no effect on TOPK or PTEN phosphorylation in H9c2 cells.

### 3.5. Oxidative Stress Indicators and Levels of TOPK, PTEN, and Akt in the Hearts of Diabetic Mice

**Figure 5:**
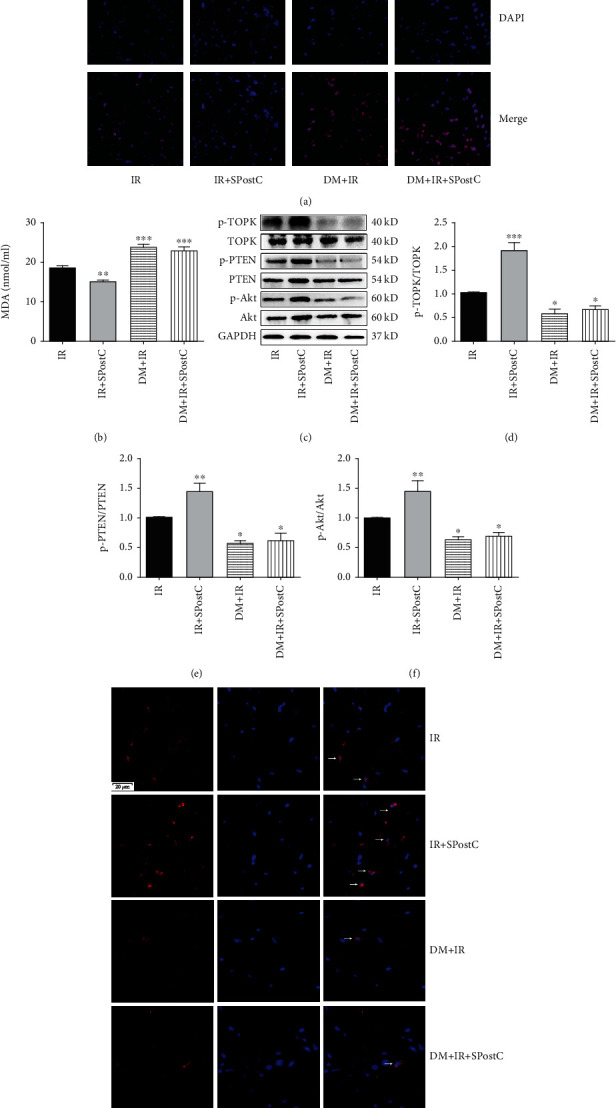
Oxidative stress and myocardial TOPK, PTEN, and Akt protein expression and their phosphorylation status assessed after myocardial IR with or without SPostC in nondiabetic and diabetic mice. (a) Representative photographs of dihydroethidium (DHE) staining detected by immunofluorescence in the mouse hearts. (DAPI: nuclei, blue; DHE fluorescence: red; magnification, ×400). (b) Serum levels of malondialdehyde (MDA) assessed by a kit. (c) Representative western blots. (d–f) Expression of TOPK (d), PTEN (e), and Akt (f) and their phosphorylation status. Mean band density was normalized relative to GAPDH. The IR group was used as control and normalized to unity, and the protein expression of other groups was displayed as changes over this baseline. (g) Myocardial phosphorylated TOPK level detected by immunofluorescence. All values are presented as the mean ± SD (*n* = 7 per group). ^∗∗^*P* < 0.01 and ^∗∗∗^*P* < 0.001 compared with the IR group.

The immunofluorescence images showed that SPostC significantly reduced myocardial superoxide anion generation, as evidenced by the decreased number of dihydroethidium labeled nuclei in the IR+SPostC group when compared with the IR group in C57BL/6 mice (Figure 5(a), *P* < 0.05). Superoxide anion accumulation was increased in the hearts of diabetic mice when compared with that in nondiabetic mice (*P* < 0.05). However, the effect of SPostC in reducing superoxide anion generation was diminished in the hearts of diabetic mice.

The level of lipid peroxidation marker MDA in the IR+SPostC group was lower than that in the IR group (Figure 5(b), *P* < 0.01). Diabetes greatly augmented serum MDA level and canceled the antilipid oxidation effect of SPostC seen in the hearts of nondiabetic mice.

The phosphorylated level of TOPK in the peri-infarct tissue was detected by immunofluorescence (Figure 5(g)). SPostC increased the amount of p-TOPK-positive cells in the hearts of nondiabetic mice. In the diabetic heart, a significant decrease in TOPK activation was observed, and SPostC exerted no effect on the phosphorylation of TOPK in the hearts of diabetic mice.

Western blot analysis showed that the phosphorylation levels of TOPK, PTEN, and Akt were significantly increased in the IR+SPostC group compared with the IR group (Figures 5(c)–5(f), *P* < 0.05). Importantly, diabetes remarkably reduced the phosphorylation levels of TOPK, PTEN, and Akt after myocardial IR (DM+IR and DM+IR+SPostC groups vs. IR group, *P* < 0.05). Nevertheless, the effect of SPostC in inducing the phosphorylation levels of TOPK, PTEN, and Akt was diminished in the hearts of diabetic mice (*P* > 0.05).

### 3.6. Posthypoxic Cellular Injury, Oxidative Stress, and Levels of TOPK, PTEN, and Akt in H9c2 Cardiomyocytes Exposed to High Glucose

**Figure 6:**
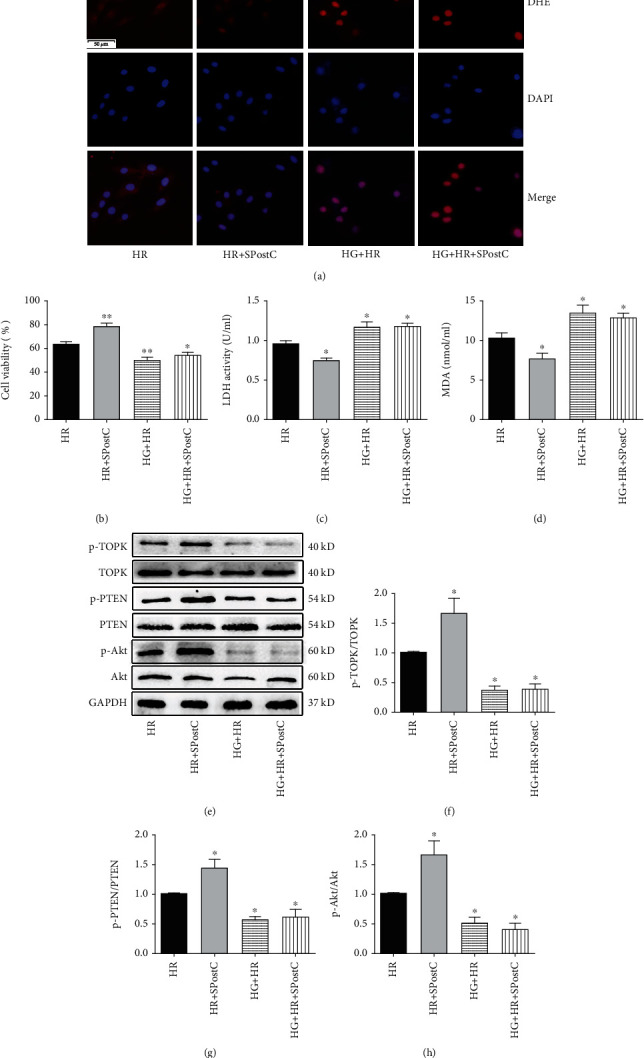
Cardiomyocyte injury and myocardial TOPK, PTEN, and Akt protein expression and their phosphorylation status as well as oxidative stress assessed after HR with or without SPostC in H9c2 cells under high glucose (HG) condition. (a) Representative photographs of dihydroethidium (DHE) staining detected by immunofluorescence in the mouse hearts. (DAPI: nuclei, blue; DHE fluorescence: red; magnification, ×400). (b) Cell viability assessed by MTT assay. (c) LDH release. (d) Serum levels of malondialdehyde (MDA) assessed by a kit. (e) Representation western blots. (f–h) Expression of TOPK (f), PTEN (g), and Akt (h) and their phosphorylation status. Mean band density was normalized relative to GAPDH. The IR group was used as control and normalized to unity, and the protein expression of other groups was displayed as changes over this baseline. All values are presented as the mean ± SD of three independent experiments each performed in triplicate. ^∗^*P* < 0.05 and ^∗∗^*P* < 0.01 compared with the HR group.

After HR injury, H9c2 cardiomyocytes exposed to high glucose showed reduced cell viability and enlarged LDH leakage when compared with cells exposed to normal glucose (Figures 6(b) and 6(c), *P* < 0.05). Moreover, the effect of SPostC in reducing posthypoxic cellular injury was abolished in cells exposed to high glucose.

Superoxide anion and MDA accumulation were increased in H9c2 cardiomyocytes exposed to high glucose as compared to the HR group (Figures 6(a) and 6(d), *P* < 0.05). The posthypoxic elevations of superoxide anion and MDA generation in cardiomyocytes were attenuated by SPostC when cells were exposed to normal glucose but not to high glucose.

Posthypoxic phosphorylated levels of TOPK, PTEN, and Akt were remarkably reduced in H9c2 cardiomyocytes exposed to high glucose as compared to the HR group (Figures 6(e)–6(h), *P* < 0.05). SPostC exerted no significant effect on the phosphorylated levels of TOPK, PTEN, and Akt in cardiomyocytes exposed to high glucose.

### 3.7. Posthypoxic Cellular Injury and Oxidative Stress in Cardiomyocytes Exposed to High Glucose after TOPK Overexpression and Levels of TOPK, PTEN, and Akt

**Figure 7:**
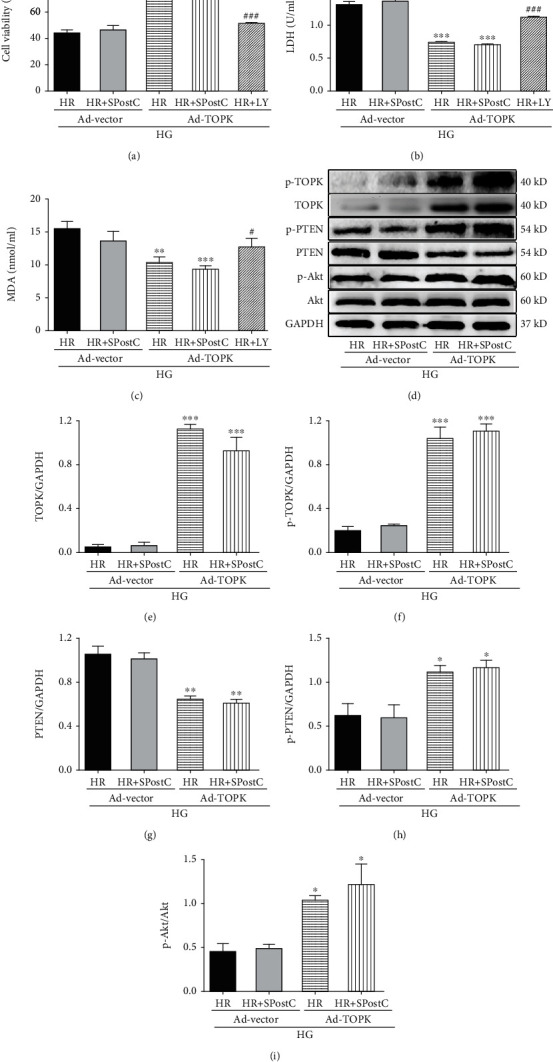
Cardiomyocyte injury, oxidative stress, and myocardial TOPK, PTEN, and Akt protein expression and their phosphorylation status assessed after HR with or without SPostC and LY in H9c2 cells under HG condition infected with adenovirus encoding rat TOPK (Ad-TOPK) or adenovirus vector (Ad-vector). (a) Cell viability was tested via MTT assay. (b) LDH release in the conditioned medium was analyzed by LDH assay. (c) MDA levels in different groups. (d) Representative western blots. Protein expression of TOPK (e, f), PTEN (g, h), and Akt (i) and their phosphorylation status in H9c2 cells detected by western blots. Mean band density was normalized relative to GAPDH. All values are presented as the mean ± SD of three independent experiments each performed in triplicate. ^∗^*P* < 0.05, ^∗∗^*P* < 0.01, and ^∗∗∗^*P* < 0.001 compared with the Ad-vector+HG+HR group; ^#^*P* < 0.05, ^##^*P* < 0.01, and compared with the Ad-TOPK+HG+HR group.

To determine whether or not cardiac TOPK overexpression can decrease the posthypoxic cellular injury in diabetes, TOPK was supplied using an adenoviral transfection system into H9c2 cells under high glucose. As shown in Figures 7(a) and 7(b), both the Ad-TOPK+HG+HR and Ad-TOPK+HG+HR+SPostC groups significantly increased cell viability and reduced LDH leakage when compared with the Ad-vector+HG+HR group (*P* < 0.001). However, when the PI3K/Akt inhibitor LY294002 was added in the Ad-TOPK+HG+HR group, the cell viability was reduced and released LDH concentration was increased significantly (*P* < 0.001), suggesting that PI3K/Akt inhibition blocked the cardioprotective effect of TOPK in H9c2 cardiomyocytes exposed to high glucose.

In order to observe the effect of TOPK supplementation on postischemic oxidative stress in diabetes, the levels of MDA and SOD were measured. Cardiac TOPK overexpression significantly decreased the postischemic MDA level (Figure 7(c)) and increased the postischemic SOD level (Figure S1(b)) in H9c2 cardiomyocytes exposed to high glucose (Ad-TOPK+HG+HR or Ad-TOPK+HG+HR+SPostC group vs. Ad-vector+HG+HR, *P* < 0.01). However, the antioxidation effect of TOPK was blocked by LY294002 in the Ad-TOPK+HG+HR+LY group when compared with the Ad-TOPK+HG+HR group (*P* < 0.05).

The bands of proteins in each group on western blot are shown in Figure 7(d). In Figure 7(e), the expression of TOPK was markedly elevated in the Ad-TOPK+HG+HR and Ad-TOPK+HG+HR+SPostC groups after TOPK transfection. The phosphorylated level of TOPK was likewise increased in the Ad-TOPK+HG+HR and Ad-TOPK+HG+HR+SPostC groups when compared with the Ad-vector+HG+HR group (Figure 7(f), *P* < 0.001). Then, the expression of PTEN was significantly reduced in the Ad-TOPK+HG+HR and Ad-TOPK+HG+HR+SPostC groups when compared with the Ad-vector+HG+HR group (Figure 7(g), *P* < 0.01). The phosphorylated levels of PTEN and Akt were enhanced in the Ad-TOPK+HG+HR and Ad-TOPK+HG+HR+SPostC groups when compared with the Ad-vector+HG+HR group (Figures 7(h) and 7(i), *P* < 0.05).

## 4. Discussion

Several major findings are presented in the current study. First, using *in vivo* mouse model of myocardial IR and *in vitro* HR model with of the H9c2 myocardial cell line, we showed that the cardioprotective effects mediated by SPostC were associated with activation of TOPK and PI3K/Akt (phosphorylated status), inactivation of PTEN (phosphorylated status), and a decrease in oxidative stress in response to myocardial IR insult. All these beneficial effects conferred by SPostC were attenuated by either the TOPK inhibitor HI-TOPK-032 or the PI3K/Akt inhibitor LY294002. The fact that TOPK inhibitor inhibited the phosphorylation of PTEN and Akt, whereas Akt inhibitor had no effect on the phosphorylation of TOPK and PTEN, it suggests that TOPK may have inactivated PTEN and in turn activated the PI3K/Akt signaling, which represents the major mechanism, whereby SPostC attenuates myocardial IR injury in nondiabetes. Second, we identified that the underlying molecular mechanism for the loss of cardioprotection of SPostC in diabetes or hyperglycemic condition is the impairment of the TOPK/PTEN/Akt signaling and the subsequently collapsed antioxidant system. In addition, TOPK supplementation protected against myocardial IR injury by activation of PTEN/Akt signaling pathway-mediated antioxidation.

SPostC has been proposed as a new strategy against myocardial IR injury, and it is more controllable, more convenient, and more conducive to clinical applications compared to ischemic postconditioning [10, 22–24]. However, the exact molecular mechanisms involved in SPostC-induced cardioprotection have not been fully clarified. TOPK, an innovative regulator of downstream PTEN/Akt pathway activity [14, 25], played a crucial role in the protection of renal or cerebral IR injury owing to its properties of antioxidation and anti-inflammation [16, 26]. PTEN, as a tumor suppressor gene, could encode a major lipid phosphatase which signals down the PI3K/Akt pathway by dephosphorylating PIP3 to PIP2, and is inactivated via phosphorylation or oxidation [27–29]. A number of studies have provided compelling evidence to confirm that the PI3K/Akt pathway was a classical signaling pathway in regulating cell proliferation, cell cycle progression, apoptosis, cell adhesion, migration, and invasion [30]. Recently, further evidence implicated that the PI3K/Akt/GSK-3*β* pathway could modulate mitochondrial dysfunction and oxidative stress and then determine the extent of myocardial IR injury [31]. Despite extensive studies, the prior reports focused primarily on how the PI3K/Akt signaling pathway influenced the cardioprotection induced by SPostC [10, 32]. It was unclear whether TOPK played critical roles in the cardioprotection of SPostC via the PTEN/Akt signaling. Herein, we demonstrate *in vivo* that the expression of TOPK in mouse cardiomyocytes after myocardial IR injury was increased, and that was further induced by SPostC. These results described above may be explained in part by the reduction of postmyocardial oxidative stress and attenuation of myocardial IR injury. Moreover, available data in our work indicated that the area of myocardial pathological injury was enlarged, the degree of cardiomyocyte apoptosis was aggravated, and the extent of cardiac myocytes injury was increased when SPostC mice were given TOPK inhibitor HI-TOPK-032 or PI3K inhibitor LY294002 beforehand. These results displayed that SPostC has a potent protective effect against myocardial IR injury possibly through phosphorylating the PTEN/ATK signaling pathway by the activation of TOPK, providing new insight into the mechanism by which SPostC reduced the risk of IR injury.

Over the course of the past 30 years, cardiovascular reperfusion therapy is still a major method for improving survival in patients suffered from acute myocardial infarction. Strikingly, several clinical studies indicated that infarct size was increased by 30–70% in diabetic patients after reperfusion therapy compared with nondiabetic patients treated in the same way, suggesting that diabetes mellitus sensitized the heart to IR injury [6, 33, 34]. As well, we found that the myocardial postischemic infarct size and apoptosis were significantly increased in diabetic mice under the treatment of 45 min ischemia followed by 2 h reperfusion, and that H9c2 cells exposed to high glucose markedly lowered its cell viability after HR as compared to H9c2 cells cultured under normal glucose. In addition, we observed that diabetes abolished the cardioprotective effect of SPostC, which fits well with previous studies which reported that diabetes may abolish the benefits of anesthetic pre/postconditioning [3, 35, 36]. Furthermore, the oxidative stress was significantly increased in both diabetic mice and H9c2 cells under high glucose, but SPostC exerted no effect on oxidative stress in diabetes [3]. Although a lot of effort has been spent on improving these weaknesses in diabetes, the efficient and effective method has yet to be developed. For example, a previous study indicated that the use of insulin for downregulating blood glucose failed to recover the advantages of SPostC, and it may be due to the diabetes-induced inhibition of the PI3K signaling [37]. Although previous studies have shown that increased cardiac PTEN is responsible for the loss of diabetic heart sensitivity to ischemic postconditioning through PI3K/Akt inactivation [9], the underlying molecular mechanism remains unknown. In our study, we found that either in streptozotocin-induced diabetic mice or in H9c2 cells exposed to high glucose, the cardioprotective effect of SPostC was canceled, accompanied by increased oxidative stress, decreased TOPK phosphorylation, decreased PTEN phosphorylation (inactivated status), and decreased Akt phosphorylation. Studies have shown that the adverse effects of hyperglycemia on grave myocardial IR injury were attributed to the oxidative stress properties [38, 39]. Therefore, these results suggested that the underlying mechanism attributable to the abolished cardioprotection induced by SPostC in diabetes or high glucose conditions is likely the impairment of the TOPK/PTEN/Akt signaling, and the subsequently collapsed antioxidant system. Then, we have extended these observations by employing gain-of-function approaches in cultured H9c2 cells to investigate the functional roles of TOPK, and initial results seem promising. Our study showed that TOPK overexpression restored posthypoxic p-PTEN and p-Akt and decreased cell death and oxidative stress in H9c2 cells exposed to high glucose, which was blocked by PI3K/Akt inhibition. Collectively, these findings indicated that TOPK supplementation prevented diabetic myocardial IR injury through PTEN/PI3K/Akt activation-mediated antioxidation.

Finally, several limitations to the present study should be considered. First, the effective activator of TOPK was not invented in practice which may limit the application of TOPK in patients with diabetic ischemic cardiomyopathy. Second, given that HI-TOPK-032 is known to be docked to the active site of TOPK to directly and specifically suppress TOPK kinase activity [12], the TOPK kinase activity was not measured after the use of HI-TOPK-032 in our current study. However, the knockdown effect of TOPK small interfering RNAs on myocardial IR injury should be studied in the nondiabetic and diabetic myocardium in the future study to further confirm the role of TOPK in the context of diabetic myocardial IR and its impact on SPostC. Third, we only discussed the benefits of SPostC conducted on acute myocardial IR injury; however, several reports demonstrated that isoflurane played a pivotal role in delayed cardioprotection [40, 41]. More considerable works on delayed cardioprotection provided by SPostC will be done in our next research work. At last, while the advantages we observed in H9c2 cells transfected with TOPK phenotype suggest that TOPK has a potential cardioprotective function in diabetes, this needs to be directly tested by measuring myocardial infarct sizes in diabetic mice, which we are currently planning.

## 5. Conclusions

**Figure 8:**
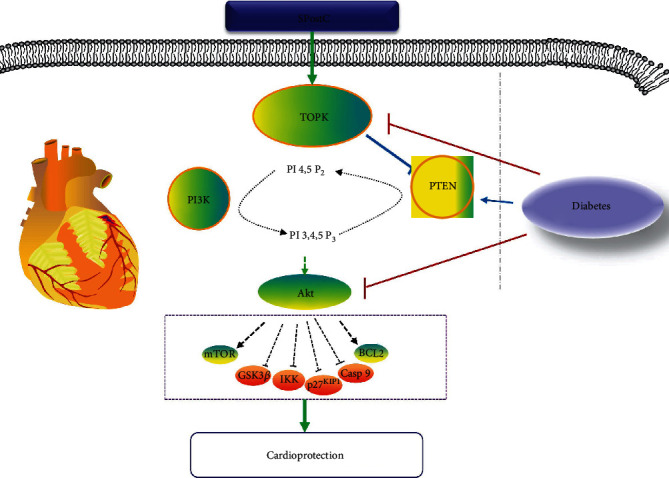
Schematic of proposed mechanism involved in the impairment of the TOPK/PTEN/Akt signaling that has rendered the diabetic hearts to loss responsiveness to sevoflurane postconditioning (SPostC) cardioprotection.

In summary, we demonstrated that SPostC protected against myocardial IR injury possibly through the activation of the TOPK/PTEN/Akt signaling pathway, while the impairment of the TOPK/PTEN/Akt signaling in cardiac might be the major mechanism that has rendered diabetic hearts less or not responsive to SPostC cardioprotection (Figure 8). In addition, TOPK supplementation protected against myocardial IR injury by activation of PTEN/Akt signaling pathway-mediated antioxidation. Our results provided new insight into the activation mechanisms of TOPK which may lead to the development of effective therapeutic targets to combat the myocardial complications of diabetes.

## Figures and Tables

**Table 1:** General characteristics.

Parameters	Sham (*n* = 7)	IR (*n* = 16)	IR+SPostC (*n* = 15)	IR+SPostC+LY (*n* = 15)	IR+SPostC+HI (*n* = 15)	DM+IR (*n* = 17)	DM+IR+SPostC (*n* = 18)
Water intake (ml/kg/day)	6.2 ± 0.8	7.2 ± 1.0	5.7 ± 1.0	7.3 ± 1.2	6.7 ± 1.4	28.4 ± 7.9^∗∗∗^	27.1 ± 4.7^∗∗∗^
Food consumption (g/kg/day)	4.6 ± 0.5	4.9 ± 0.5	4.3 ± 0.6	4.7 ± 0.7	5.0 ± 0.5	6.6 ± 1.0^∗∗∗^	6.4 ± 0.7^∗∗∗^
Body weight (g)	27.6 ± 0.9	25.9 ± 1.6	26.6 ± 1.8	26.3 ± 1.3	26.3 ± 1.4	23.2 ± 1.0^∗∗∗^	23.3 ± 1.0^∗∗∗^
Plasma glucose (mM)	5.7 ± 0.7	6.1 ± 0.6	6.4 ± 0.6	5.9 ± 0.5	6.0 ± 0.5	26.0 ± 4.0^∗∗∗^	24.9 ± 4.6^∗∗∗^

Nondiabetic and diabetic mice (DM) were subjected to myocardial ischemia/reperfusion (IR) with or without sevoflurane postconditioning (SPostC) in the presence or absence of the PI3K inhibitor LY294002 (LY) or the TOPK kinase inhibitor HI-TOPK-032 (HI). All values are expressed as the mean ± SD. Water intake and food consumption were the average value of 8 weeks. Body weight and plasma glucose were measured among groups before inducing myocardial IR. ^∗∗∗^*P* < 0.001 vs. their corresponding IR groups.

**Table 2:** Hemodynamic measurements at baseline, at 15 min of ischemia, and at 2 h of reperfusion in nondiabetic and diabetic rats with or without treatments.

Parameters	Sham	IR	IR+SPostC	IR+SPostC+LY	IR+SPostC+HI	DM+IR	DM+IR+SPostC
*Heart rate (beats/min)*
Baseline	368 ± 9	370 ± 12	378 ± 14	371 ± 12	378 ± 14	384 ± 15	383 ± 16
Ischemia 15 min	375 ± 9	372 ± 14	369 ± 8	370 ± 12	366 ± 12	370 ± 15	370 ± 12
Reperfusion 2 h	373 ± 8	368 ± 11	368 ± 8	366 ± 17	370 ± 11	368 ± 16	365 ± 17
*Mean arterial blood pressure (mmHg)*
Baseline	82.9 ± 5.1	79.8 ± 5.6	81.9 ± 4.5	79.3 ± 5.5	78.3 ± 4.5	79.7 ± 5.7	81.2 ± 4.7
Ischemia 15 min	81.3 ± 6.2	62.1 ± 4.7^∗∗^	62.7 ± 5.3^∗∗^	63.4 ± 5.9^∗∗^	62.1 ± 4.8^∗∗^	55.1 ± 4.3^∗∗#^	53.9 ± 5.1^∗∗#^
Reperfusion 2 h	83.0 ± 5.8	59.3 ± 2.3^∗∗^	66 ± 5.4^∗∗#^	60.6 ± 3.4^∗∗^	60 ± 3.7^∗∗^	53.0 ± 5.3^∗∗#^	50.9 ± 5.1^∗∗##^

Nondiabetic and diabetic mice (DM) were subjected to myocardial ischemia/reperfusion (IR) with or without sevoflurane postconditioning (SPostC) in the presence or absence of the PI3K inhibitor LY294002 (LY) or the TOPK kinase inhibitor HI-TOPK-032 (HI). All values are expressed as the mean ± SD. *n* = 7 per group. Heart rate and mean arterial pressure were measured at baseline and during myocardial IR. ^∗∗^*P* < 0.01 vs. their corresponding baseline; ^#^*P* < 0.05 and ^##^*P* < 0.01 vs. their corresponding IR groups.

## Data Availability

The data used to support the findings of this study are available from the corresponding authors upon request.

## Abbreviations

TOPK: T-LAK cell-originated protein kinase
SPostC: Sevoflurane postconditioning
PTEN: Phosphatase and tensin homolog deleted on chromosome ten
PI3K: Phosphatidylinositol 3-kinase
Akt: Protein kinase B
IR: Ischemia/reperfusion
MAPKK: Mitogen-activated protein kinase
CK-MB: Creatine kinase-MB
TUNEL: Terminal deoxynucleotidyl transferase-mediated nick end labeling
MDA: Malondialdehyde
LDH: Lactate dehydrogenase
PIP3: Phosphatidylinositol 3,4,5-triphosphate
PIP2: Phosphatidylinositol 4,5-biphosphate
GSK-3*β*: Glycogen synthase kinase-3*β*.

## Additional Points

(i) Sevoflurane postconditioning protected the heart from ischemia/reperfusion injury possibly through TOPK-mediated PTEN/PI3K/Akt activation in nondiabetic but not in diabetic rodents. (ii) Diabetes blocked the cardioprotection of sevoflurane postconditioning by impairing TOPK bioavailability. (iii) TOPK supplementation prevented diabetic myocardial ischemia/reperfusion injury through PTEN/PI3K/Akt activation

## Supplementary Materials

Supplementary Materials Figure S1: (a) representative images of Evans blue and TTC staining in heart cross sections from the sham group. (b) SOD level assessed after HR with or without SPostC and LY294002 (LY) in H9c2 cells under HG condition infected with adenovirus encoding rat TOPK (Ad-TOPK) or adenovirus vector (Ad-vector). ^∗∗∗^*P* < 0.001 compared with the Ad-vector+HG+HR group; ^###^*P* < 0.001 compared with the Ad-TOPK+HG+HR group.
