# Supplementary material for: Inactivation of TOPK Caused by Hyperglycemia Blocks Diabetic Heart Sensitivity to Sevoflurane Postconditioning by Impairing the PTEN/PI3K/Akt Signaling
Source: Oxid Med Cell Longev. 2021 Apr 23;2021:6657529. doi: 10.1155/2021/6657529 (PMC8093075; doi:10.1155/2021/6657529)
Supplement: Supplementary Materials — Figure S1: (a) representative images of Evans blue and TTC staining in heart cross sections from the sham group. (b) SOD level assessed after HR with or without SPostC and LY294002 (LY) in H9c2 cells under HG condition infected with adenovirus encoding rat TOPK (Ad-TOPK) or adenovirus vector (Ad-vector). ∗∗∗P < 0.001 compared with the Ad-vector+HG+HR group; ###P < 0.001 compared with the Ad-TOPK+HG+HR group. [file 6657529.f1.docx]

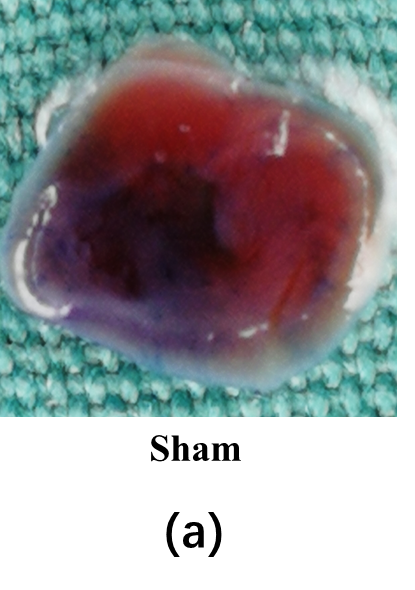

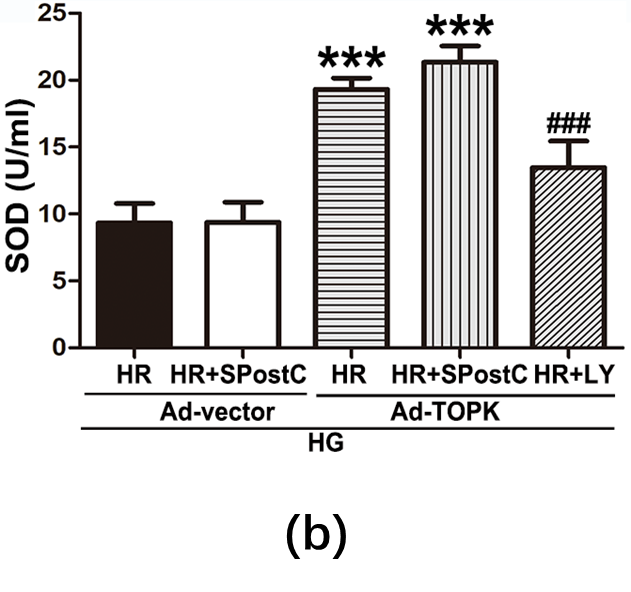


Fig. S1: (a) Representative images of Evans Blue and TTC staining in heart cross-sections from sham group. (b) SOD level assessed after HR with or without SPostC and LY294002 (LY) in H9c2 cells under HG condition infected with adenovirus encoding rat TOPK (Ad-TOPK) or adenovirus vector (Ad-vector). All values are presented as mean ± S.D. of three independent experiments each performed in triplicate. ***P<0.001 compared with Ad-vector+HG+HR group; ###P<0.001 compared with Ad-TOPK+HG+HR group.
